# *catena*-Poly[1-butyl-3-methyl­imidazolium [[dichlorido(methanol-κ*O*)(propan-2-ol-κ*O*)lanthanate(III)]-di-μ-chlorido]]

**DOI:** 10.1107/S160053681200517X

**Published:** 2012-02-17

**Authors:** Yulun Han, Fengrong Dai, Andrew G. Sykes, P. Stanley May, Mary T. Berry, Qingguo Meng, Cuikun Lin

**Affiliations:** a Department of Chemistry, University of South Dakota, 414E Clark, CL115, Vermillion, SD 57069, USA

## Abstract

The title compound, (C_8_H_15_N_2_)[LaCl_4_(CH_3_OH)(C_3_H_7_OH)], consists of one 1-butyl-3-methyl­imidazolium (BMI^+^) cation and one hexa­hedral tetra­chlorido(methanol)(propan-2-ol)lanthanate anion. The La^III^ ion is eight-coordinate, with the La^III^ ion bridged by a pair of Cl atoms, so forming chains propagating along the *a*-axis direction. Each La^III^ ion is further coordinated by two isolated Cl atoms, one methanol and one propan-2-ol mol­ecule. The coordinated methanol and propan-2-ol mol­ecules of the anion form O—H⋯Cl hydrogen bonds with the Cl atoms of inversion-related anions. The BMI^+^ cation froms C—H⋯Cl hydrogen bonds with the Cl atoms of the anion. The anions are located in the C faces of the triclinic unit cell, with an inversion center in the middle of the La_2_Cl_2_ ring of the polymeric chain.

## Related literature

For related crystal structures, see: Binnemans (2007 ▶); Pellens *et al.* (2008 ▶); Matsumoto *et al.* (2002 ▶). For the synthesis of the title compound, see: Burrell *et al.* (2007 ▶). For the optical properties of lanthanides in ionic liquids, see: Brandner *et al.* (2011 ▶); Samikkanu *et al.* (2007 ▶).
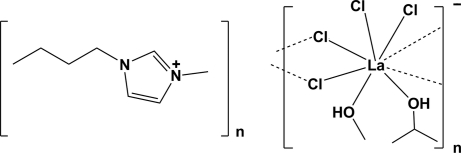


## Experimental

### 

#### Crystal data

(C_8_H_15_N_2_)[LaCl_4_(CH_4_O)(C_3_H_8_O)]
*M* *_r_* = 512.07
Triclinic, 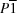

*a* = 9.5035 (6) Å
*b* = 10.7413 (6) Å
*c* = 11.8625 (7) Å
α = 114.009 (1)°
β = 109.735 (1)°
γ = 92.857 (1)°
*V* = 1016.20 (10) Å^3^
*Z* = 2
Mo *K*α radiation
μ = 2.63 mm^−1^
*T* = 100 K
0.30 × 0.15 × 0.05 mm

#### Data collection

Bruker APEXII CCD area-detector diffractometer
Absorption correction: multi-scan (*SADABS*; Sheldrick, 1996 ▶) *T*_min_ = 0.506, *T*_max_ = 0.880
11178 measured reflections
4155 independent reflections
3780 reflections with *I* > 2σ(*I*)
*R*_int_ = 0.029

#### Refinement

*R*[*F* ^2^ > 2σ(*F* ^2^)] = 0.022
*wR*(*F* ^2^) = 0.049
*S* = 1.04
4155 reflections
198 parameters
H atoms treated by a mixture of independent and constrained refinement
Δρ_max_ = 0.71 e Å^−3^
Δρ_min_ = −0.59 e Å^−3^

### 

Data collection: *APEX2* (Bruker, 2007 ▶); cell refinement: *SAINT* (Bruker, 2007 ▶); data reduction: *SAINT*; program(s) used to solve structure: *SIR92* (Altomare *et al.*, 1994 ▶); program(s) used to refine structure: *SHELXL97* (Sheldrick, 2008 ▶); molecular graphics: *Mercury* (Macrae *et al.*, 2008 ▶); software used to prepare material for publication: *publCIF* (Westrip, 2010 ▶).

## Supplementary Material

Crystal structure: contains datablock(s) I, global. DOI: 10.1107/S160053681200517X/su2369sup1.cif

Structure factors: contains datablock(s) I. DOI: 10.1107/S160053681200517X/su2369Isup2.hkl

Additional supplementary materials:  crystallographic information; 3D view; checkCIF report

## Figures and Tables

**Table 1:** Hydrogen-bond geometry (Å, °)

*D*—H⋯*A*	*D*—H	H⋯*A*	*D*⋯*A*	*D*—H⋯*A*
O1—H1*D*⋯Cl2^i^	0.79 (4)	2.42 (4)	3.206 (2)	171 (4)
O2—H2*B*⋯Cl1^ii^	0.75 (4)	2.39 (4)	3.122 (2)	166 (4)
C5—H5*A*⋯Cl3^iii^	0.93	2.65	3.458 (3)	145
C8—H8*A*⋯Cl3^iii^	0.96	2.67	3.565 (3)	156

Symmetry codes: (i) 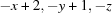; (ii) 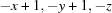; (iii) 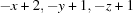.

**Fig. 1.**
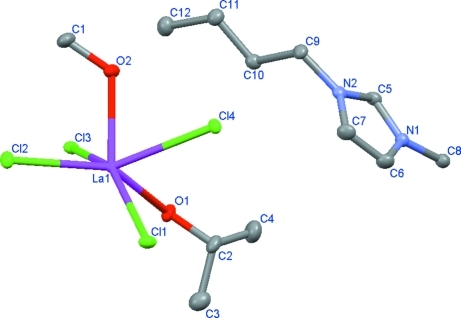
The molecular structure of the asymmetric unit of the title compound, with the numbering scheme and displacement ellipsoids drawn at the 50% probability level. H atoms have been omitted for clarify.

**Fig. 2.**
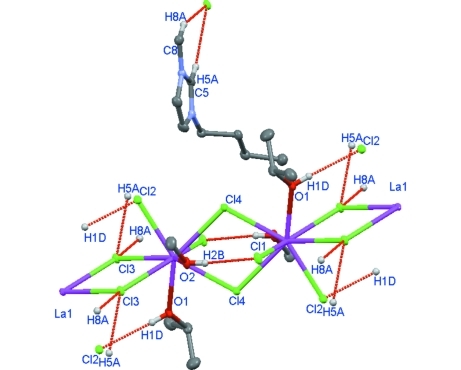
A view of the molecular structure of the title compound, with the dashed lines denoting the hydrogen bonding.

**Fig. 3.**
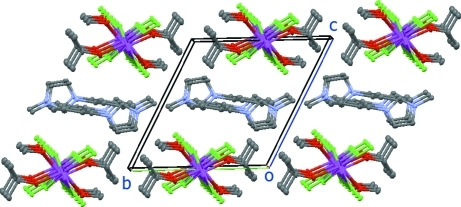
Crystal packing of the title compound viewed along the a axis. The [LaCl4(CH3OH)(i-C3H9OH)]- anions are located about the inversion centers in the C faces of the triclinic unit cell.

## supplementary crystallographic information

### Comment

Ionic liquids (ILs) have received considerable attention due to their extraordinary properties as solvents (Binnemans, 2007). They have been proposed as excellent alternatives to conventional solvents for luminescent lanthanide complexes. Compared with aqueous or organic solvents, ILs have the advantages of potentially excluding quenching oscillators, providing greater luminescence quantum yields (Brandner *et al.*, 2011; Samikkanu *et al.*, 2007). Some analogous structures to the title compound, tris(1-ethyl-3-methylimidazolium)hexabromidoeuropate(III) (Pellens *et al.*, 2008) and tris(1-ethyl-3-methylimidazolium)hexachloridolanthanate(III) (Matsumoto *et al.*, 2002) have been reported.

The title compound, in contrast to these examples, includes coordinated alcohol molecules and crystallized after mixing lanthanum chloride in 1-butyl-3-methylimidazolium chloride (BMICl) with a mixture of methanol and propan-2-ol (Fig. 1). The bond lengths between La and the two non-bridging Cl atoms are 2.8232 (5) Å and 2.838 (1) Å, respectively. The La to bridging Cl distances are in the range of 2.8884 (6) Å and 3.0021 (8) Å. All the Cl atoms, except Cl4, exhibit short contacts to neighboring H atoms on the imidazolium rings or on alcohol molecules ranging from 2.653 Å to 2.909 Å.

In the crystal, H atoms in the imidazolium cations, such as H5A and H8A, form hydrogen bonds with chlorine Cl3 (Fig. 2 and Table 1). The two H atoms in methanol (H2B) and propan-2-ol (H1D) form hydrogen bonds with atoms Cl2 and Cl1, respectively (Table 1). The [LaCl_4_(CH_3_OH)(*i*-C_3_H_9_OH)]^-^ anions are centered in the C faces of the triclinic unit cell, with an inversion center in the middle of La_2_Cl_2_ ring, as shown in Fig. 3. The BMI^+^ cation is on an inversion center, at position (1/2, 1/2, 1/2) in the unit cell.

### Experimental

1-butyl-3-methylimidazolium chloride (BMICl) was synthesized following a method reported by Burrell *et al.* (2007). Lanthanum chloride heptahydrate (0.708 g, 1.906 mmol) was mixed with BMICl (1.000 g, 5.725 mmol) in a small vial in a glove box. Equal amount of methanol and propan-2-ol were added carefully until the total dissolution of the mixture. The vial was sealed and a colourless crystal appeared after cooling at 258 K for three weeks.

### Refinement

The OH H atoms were located in a difference Fourier map and were freely refined. The C-bound H atoms were included in calculated positions and treated as riding atoms: C—H = 0.98, 0.97 and 0.96 Å for CH, CH_2_ and CH_3_ H-atoms, respectively, with *U*_iso_(H) = k × *U*_eq_(parent C-atom), where k = 1.5 for CH_3_ H atoms and k = 1.2 for all other H atoms.

### Crystal data

**Table:**

(C_8_H_15_N_2_)[LaCl_4_(CH_4_O)(C_3_H_8_O)]	*Z* = 2
*M**_r_* = 512.07	*F*(000) = 508
Triclinic, *P*1	*D*_x_ = 1.674 Mg m^−^^3^
Hall symbol: -P 1	Mo *K*α radiation, λ = 0.71073 Å
*a* = 9.5035 (6) Å	Cell parameters from 8208 reflections
*b* = 10.7413 (6) Å	θ = 2.3–26.4°
*c* = 11.8625 (7) Å	µ = 2.63 mm^−^^1^
α = 114.009 (1)°	*T* = 100 K
β = 109.735 (1)°	Block, colourless
γ = 92.857 (1)°	0.30 × 0.15 × 0.05 mm
*V* = 1016.20 (10) Å^3^	

### Data collection

**Table:**

Bruker APEXII CCD area-detector diffractometer	4155 independent reflections
Radiation source: fine-focus sealed tube	3780 reflections with *I* > 2σ(*I*)
Graphite monochromator	*R*_int_ = 0.029
φ and ω scans	θ_max_ = 26.4°, θ_min_ = 2.0°
Absorption correction: multi-scan (*SADABS*; Sheldrick, 1996)	*h* = −11→11
*T*_min_ = 0.506, *T*_max_ = 0.880	*k* = −13→13
11178 measured reflections	*l* = −14→14

### Refinement

**Table:**

Refinement on *F*^2^	Primary atom site location: structure-invariant direct methods
Least-squares matrix: full	Secondary atom site location: difference Fourier map
*R*[*F*^2^ > 2σ(*F*^2^)] = 0.022	Hydrogen site location: inferred from neighbouring sites
*wR*(*F*^2^) = 0.049	H atoms treated by a mixture of independent and constrained refinement
*S* = 1.04	*w* = 1/[σ^2^(*F*_o_^2^) + (0.0193*P*)^2^ + 0.4077*P*] where *P* = (*F*_o_^2^ + 2*F*_c_^2^)/3
4155 reflections	(Δ/σ)_max_ = 0.001
198 parameters	Δρ_max_ = 0.71 e Å^−^^3^
0 restraints	Δρ_min_ = −0.59 e Å^−^^3^

### Special details

**Table:**

Geometry. All e.s.d.'s (except the e.s.d. in the dihedral angle between two l.s. planes) are estimated using the full covariance matrix. The cell e.s.d.'s are taken into account individually in the estimation of e.s.d.'s in distances, angles and torsion angles; correlations between e.s.d.'s in cell parameters are only used when they are defined by crystal symmetry. An approximate (isotropic) treatment of cell e.s.d.'s is used for estimating e.s.d.'s involving l.s. planes.
Refinement. Refinement of *F*^2^ against ALL reflections. The weighted *R*-factor *wR* and goodness of fit *S* are based on *F*^2^, conventional *R*-factors *R* are based on *F*, with *F* set to zero for negative *F*^2^. The threshold expression of *F*^2^ > σ(*F*^2^) is used only for calculating *R*-factors(gt) *etc*. and is not relevant to the choice of reflections for refinement. *R*-factors based on *F*^2^ are statistically about twice as large as those based on *F*, and *R*-factors based on ALL data will be even larger.

### Fractional atomic coordinates and isotropic or equivalent isotropic displacement parameters (Å^2^)

**Table:**

	*x*	*y*	*z*	*U*_iso_*/*U*_eq_	
La1	0.739307 (15)	0.491237 (15)	−0.023339 (14)	0.00964 (5)	
Cl1	0.54404 (7)	0.24123 (7)	−0.24214 (6)	0.01713 (14)	
Cl2	0.77665 (7)	0.64602 (7)	−0.16020 (7)	0.01686 (14)	
Cl3	1.05144 (7)	0.63430 (7)	0.15869 (6)	0.01356 (13)	
Cl4	0.56003 (7)	0.42798 (7)	0.11059 (6)	0.01393 (13)	
O1	0.8605 (2)	0.3297 (2)	0.0650 (2)	0.0176 (4)	
H1D	0.951 (4)	0.344 (4)	0.096 (4)	0.042 (11)*	
O2	0.7406 (2)	0.7145 (2)	0.16709 (19)	0.0169 (4)	
H2B	0.668 (4)	0.710 (4)	0.180 (4)	0.040 (12)*	
N1	0.6581 (2)	0.1728 (2)	0.4667 (2)	0.0145 (5)	
N2	0.6359 (2)	0.3591 (2)	0.4388 (2)	0.0148 (5)	
C1	0.7807 (3)	0.8505 (3)	0.1784 (3)	0.0253 (7)	
H1A	0.7734	0.9196	0.2577	0.038*	
H1B	0.7118	0.8565	0.1010	0.038*	
H1C	0.8837	0.8666	0.1841	0.038*	
C2	0.7981 (3)	0.1900 (3)	0.0376 (3)	0.0218 (6)	
H2A	0.6864	0.1766	0.0048	0.026*	
C3	0.8374 (3)	0.0814 (3)	−0.0713 (3)	0.0297 (7)	
H3A	0.7993	0.0941	−0.1509	0.045*	
H3B	0.7911	−0.0104	−0.0907	0.045*	
H3C	0.9465	0.0918	−0.0409	0.045*	
C4	0.8534 (4)	0.1779 (4)	0.1663 (3)	0.0335 (8)	
H4A	0.8255	0.2488	0.2310	0.050*	
H4B	0.9628	0.1897	0.2001	0.050*	
H4C	0.8073	0.0875	0.1502	0.050*	
C5	0.6982 (3)	0.3114 (3)	0.5278 (3)	0.0152 (6)	
H5A	0.7599	0.3662	0.6180	0.018*	
C6	0.5659 (3)	0.1306 (3)	0.3339 (3)	0.0188 (6)	
H6A	0.5214	0.0389	0.2686	0.023*	
C7	0.5522 (3)	0.2461 (3)	0.3163 (3)	0.0195 (6)	
H7A	0.4967	0.2492	0.2365	0.023*	
C8	0.7080 (3)	0.0804 (3)	0.5284 (3)	0.0168 (6)	
H8A	0.7713	0.1356	0.6219	0.025*	
H8B	0.7654	0.0220	0.4847	0.025*	
H8C	0.6199	0.0232	0.5191	0.025*	
C9	0.6552 (3)	0.5071 (3)	0.4667 (3)	0.0167 (6)	
H9A	0.6495	0.5616	0.5526	0.020*	
H9B	0.5712	0.5167	0.3986	0.020*	
C10	0.8046 (3)	0.5662 (3)	0.4693 (3)	0.0175 (6)	
H10A	0.8113	0.5136	0.3833	0.021*	
H10B	0.8899	0.5585	0.5378	0.021*	
C11	0.8133 (3)	0.7191 (3)	0.4988 (3)	0.0199 (6)	
H11A	0.8119	0.7717	0.5869	0.024*	
H11B	0.7238	0.7264	0.4335	0.024*	
C12	0.9580 (3)	0.7831 (3)	0.4944 (3)	0.0250 (7)	
H12A	0.9588	0.8790	0.5135	0.038*	
H12B	0.9588	0.7325	0.4068	0.038*	
H12C	1.0469	0.7778	0.5601	0.038*	

### Atomic displacement parameters (Å^2^)

**Table:**

	*U*^11^	*U*^22^	*U*^33^	*U*^12^	*U*^13^	*U*^23^
La1	0.00730 (8)	0.00983 (8)	0.01066 (8)	0.00150 (5)	0.00309 (6)	0.00398 (6)
Cl1	0.0115 (3)	0.0139 (3)	0.0183 (3)	0.0012 (2)	0.0051 (3)	0.0010 (3)
Cl2	0.0131 (3)	0.0205 (4)	0.0221 (3)	0.0054 (3)	0.0073 (3)	0.0138 (3)
Cl3	0.0089 (3)	0.0143 (3)	0.0129 (3)	0.0018 (2)	0.0032 (2)	0.0029 (3)
Cl4	0.0113 (3)	0.0179 (3)	0.0158 (3)	0.0046 (2)	0.0060 (2)	0.0099 (3)
O1	0.0107 (10)	0.0165 (10)	0.0292 (11)	0.0036 (8)	0.0068 (9)	0.0143 (9)
O2	0.0120 (10)	0.0131 (10)	0.0206 (11)	−0.0001 (8)	0.0072 (8)	0.0027 (8)
N1	0.0128 (11)	0.0147 (12)	0.0153 (11)	0.0024 (9)	0.0057 (9)	0.0061 (10)
N2	0.0118 (11)	0.0168 (12)	0.0173 (12)	0.0046 (9)	0.0077 (9)	0.0073 (10)
C1	0.0278 (16)	0.0136 (15)	0.0324 (18)	0.0036 (12)	0.0152 (14)	0.0059 (13)
C2	0.0188 (14)	0.0170 (15)	0.0346 (17)	0.0055 (12)	0.0112 (13)	0.0156 (14)
C3	0.0246 (16)	0.0244 (17)	0.0350 (19)	0.0063 (13)	0.0079 (14)	0.0117 (15)
C4	0.041 (2)	0.0298 (19)	0.038 (2)	0.0069 (15)	0.0174 (16)	0.0215 (16)
C5	0.0124 (13)	0.0172 (14)	0.0124 (13)	0.0032 (11)	0.0039 (10)	0.0042 (11)
C6	0.0169 (14)	0.0161 (15)	0.0153 (14)	0.0017 (11)	0.0034 (11)	0.0023 (12)
C7	0.0173 (14)	0.0205 (15)	0.0132 (14)	0.0036 (11)	0.0013 (11)	0.0045 (12)
C8	0.0179 (14)	0.0158 (14)	0.0165 (14)	0.0034 (11)	0.0080 (11)	0.0061 (12)
C9	0.0163 (14)	0.0162 (14)	0.0207 (15)	0.0066 (11)	0.0081 (11)	0.0102 (12)
C10	0.0136 (13)	0.0188 (15)	0.0173 (14)	0.0029 (11)	0.0052 (11)	0.0065 (12)
C11	0.0174 (14)	0.0189 (15)	0.0231 (15)	0.0046 (11)	0.0066 (12)	0.0101 (13)
C12	0.0231 (15)	0.0249 (17)	0.0271 (17)	0.0023 (13)	0.0089 (13)	0.0128 (14)

### Geometric parameters (Å, º)

**Table:**

La1—O1	2.5102 (19)	C3—H3A	0.9600
La1—O2	2.5348 (19)	C3—H3B	0.9600
La1—Cl1	2.8232 (6)	C3—H3C	0.9600
La1—Cl2	2.8378 (7)	C4—H4A	0.9600
La1—Cl3	2.8884 (6)	C4—H4B	0.9600
La1—Cl4	2.9119 (6)	C4—H4C	0.9600
La1—Cl4^i^	2.9841 (6)	C5—H5A	0.9300
La1—Cl3^ii^	3.0021 (6)	C6—C7	1.346 (4)
Cl3—La1^ii^	3.0021 (6)	C6—H6A	0.9300
Cl4—La1^i^	2.9841 (6)	C7—H7A	0.9300
O1—C2	1.446 (3)	C8—H8A	0.9600
O1—H1D	0.79 (4)	C8—H8B	0.9600
O2—C1	1.431 (3)	C8—H8C	0.9600
O2—H2B	0.75 (4)	C9—C10	1.512 (4)
N1—C5	1.329 (3)	C9—H9A	0.9700
N1—C6	1.378 (3)	C9—H9B	0.9700
N1—C8	1.466 (3)	C10—C11	1.525 (4)
N2—C5	1.332 (3)	C10—H10A	0.9700
N2—C7	1.381 (3)	C10—H10B	0.9700
N2—C9	1.474 (3)	C11—C12	1.533 (4)
C1—H1A	0.9600	C11—H11A	0.9700
C1—H1B	0.9600	C11—H11B	0.9700
C1—H1C	0.9600	C12—H12A	0.9600
C2—C4	1.500 (4)	C12—H12B	0.9600
C2—C3	1.520 (4)	C12—H12C	0.9600
C2—H2A	0.9800		
			
O1—La1—O2	110.49 (6)	C3—C2—H2A	107.9
O1—La1—Cl1	83.72 (5)	C2—C3—H3A	109.5
O2—La1—Cl1	142.48 (5)	C2—C3—H3B	109.5
O1—La1—Cl2	141.74 (5)	H3A—C3—H3B	109.5
O2—La1—Cl2	89.42 (5)	C2—C3—H3C	109.5
Cl1—La1—Cl2	100.30 (2)	H3A—C3—H3C	109.5
O1—La1—Cl3	72.67 (5)	H3B—C3—H3C	109.5
O2—La1—Cl3	70.43 (5)	C2—C4—H4A	109.5
Cl1—La1—Cl3	146.015 (18)	C2—C4—H4B	109.5
Cl2—La1—Cl3	84.745 (19)	H4A—C4—H4B	109.5
O1—La1—Cl4	72.72 (5)	C2—C4—H4C	109.5
O2—La1—Cl4	70.12 (5)	H4A—C4—H4C	109.5
Cl1—La1—Cl4	82.364 (19)	H4B—C4—H4C	109.5
Cl2—La1—Cl4	145.479 (18)	N1—C5—N2	109.0 (2)
Cl3—La1—Cl4	112.230 (18)	N1—C5—H5A	125.5
O1—La1—Cl4^i^	141.39 (5)	N2—C5—H5A	125.5
O2—La1—Cl4^i^	71.22 (5)	C7—C6—N1	107.5 (2)
Cl1—La1—Cl4^i^	76.418 (18)	C7—C6—H6A	126.3
Cl2—La1—Cl4^i^	75.142 (17)	N1—C6—H6A	126.3
Cl3—La1—Cl4^i^	136.524 (19)	C6—C7—N2	107.1 (2)
Cl4—La1—Cl4^i^	72.078 (19)	C6—C7—H7A	126.5
O1—La1—Cl3^ii^	70.16 (5)	N2—C7—H7A	126.5
O2—La1—Cl3^ii^	139.40 (5)	N1—C8—H8A	109.5
Cl1—La1—Cl3^ii^	77.752 (18)	N1—C8—H8B	109.5
Cl2—La1—Cl3^ii^	73.582 (18)	H8A—C8—H8B	109.5
Cl3—La1—Cl3^ii^	71.48 (2)	N1—C8—H8C	109.5
Cl4—La1—Cl3^ii^	139.352 (18)	H8A—C8—H8C	109.5
Cl4^i^—La1—Cl3^ii^	134.605 (17)	H8B—C8—H8C	109.5
La1—Cl3—La1^ii^	108.52 (2)	N2—C9—C10	113.7 (2)
La1—Cl4—La1^i^	107.922 (19)	N2—C9—H9A	108.8
C2—O1—La1	130.59 (16)	C10—C9—H9A	108.8
C2—O1—H1D	109 (3)	N2—C9—H9B	108.8
La1—O1—H1D	118 (3)	C10—C9—H9B	108.8
C1—O2—La1	123.48 (17)	H9A—C9—H9B	107.7
C1—O2—H2B	108 (3)	C9—C10—C11	109.5 (2)
La1—O2—H2B	112 (3)	C9—C10—H10A	109.8
C5—N1—C6	108.2 (2)	C11—C10—H10A	109.8
C5—N1—C8	125.9 (2)	C9—C10—H10B	109.8
C6—N1—C8	125.8 (2)	C11—C10—H10B	109.8
C5—N2—C7	108.2 (2)	H10A—C10—H10B	108.2
C5—N2—C9	125.7 (2)	C10—C11—C12	112.2 (2)
C7—N2—C9	126.1 (2)	C10—C11—H11A	109.2
O2—C1—H1A	109.5	C12—C11—H11A	109.2
O2—C1—H1B	109.5	C10—C11—H11B	109.2
H1A—C1—H1B	109.5	C12—C11—H11B	109.2
O2—C1—H1C	109.5	H11A—C11—H11B	107.9
H1A—C1—H1C	109.5	C11—C12—H12A	109.5
H1B—C1—H1C	109.5	C11—C12—H12B	109.5
O1—C2—C4	108.9 (2)	H12A—C12—H12B	109.5
O1—C2—C3	110.9 (2)	C11—C12—H12C	109.5
C4—C2—C3	113.2 (3)	H12A—C12—H12C	109.5
O1—C2—H2A	107.9	H12B—C12—H12C	109.5
C4—C2—H2A	107.9		
			
O1—La1—Cl3—La1^ii^	74.31 (5)	Cl1—La1—O2—C1	−116.57 (19)
O2—La1—Cl3—La1^ii^	−165.68 (5)	Cl2—La1—O2—C1	−10.28 (19)
Cl1—La1—Cl3—La1^ii^	26.22 (4)	Cl3—La1—O2—C1	74.35 (19)
Cl2—La1—Cl3—La1^ii^	−74.42 (2)	Cl4—La1—O2—C1	−161.9 (2)
Cl4—La1—Cl3—La1^ii^	136.68 (2)	Cl4^i^—La1—O2—C1	−84.77 (19)
Cl4^i^—La1—Cl3—La1^ii^	−136.31 (2)	Cl3^ii^—La1—O2—C1	53.2 (2)
Cl3^ii^—La1—Cl3—La1^ii^	0.0	La1—O1—C2—C4	−139.1 (2)
O1—La1—Cl4—La1^i^	−163.89 (5)	La1—O1—C2—C3	95.8 (2)
O2—La1—Cl4—La1^i^	75.95 (5)	C6—N1—C5—N2	−0.4 (3)
Cl1—La1—Cl4—La1^i^	−78.13 (2)	C8—N1—C5—N2	177.5 (2)
Cl2—La1—Cl4—La1^i^	18.96 (4)	C7—N2—C5—N1	0.3 (3)
Cl3—La1—Cl4—La1^i^	133.77 (2)	C9—N2—C5—N1	−178.9 (2)
Cl4^i^—La1—Cl4—La1^i^	0.0	C5—N1—C6—C7	0.4 (3)
Cl3^ii^—La1—Cl4—La1^i^	−139.14 (2)	C8—N1—C6—C7	−177.5 (2)
O2—La1—O1—C2	119.5 (2)	N1—C6—C7—N2	−0.2 (3)
Cl1—La1—O1—C2	−24.7 (2)	C5—N2—C7—C6	0.0 (3)
Cl2—La1—O1—C2	−123.36 (19)	C9—N2—C7—C6	179.1 (2)
Cl3—La1—O1—C2	−179.9 (2)	C5—N2—C9—C10	80.0 (3)
Cl4—La1—O1—C2	59.3 (2)	C7—N2—C9—C10	−99.1 (3)
Cl4^i^—La1—O1—C2	34.2 (2)	N2—C9—C10—C11	−180.0 (2)
Cl3^ii^—La1—O1—C2	−103.9 (2)	C9—C10—C11—C12	−176.5 (2)
O1—La1—O2—C1	136.28 (19)		

Symmetry codes: (i) −*x*+1, −*y*+1, −*z*; (ii) −*x*+2, −*y*+1, −*z*.

### Hydrogen-bond geometry (Å, º)

**Table:**

*D*—H···*A*	*D*—H	H···*A*	*D*···*A*	*D*—H···*A*
O1—H1*D*···Cl2^ii^	0.79 (4)	2.42 (4)	3.206 (2)	171 (4)
O2—H2*B*···Cl1^i^	0.75 (4)	2.39 (4)	3.122 (2)	166 (4)
C5—H5*A*···Cl3^iii^	0.93	2.65	3.458 (3)	145
C8—H8*A*···Cl3^iii^	0.96	2.67	3.565 (3)	156

Symmetry codes: (i) −*x*+1, −*y*+1, −*z*; (ii) −*x*+2, −*y*+1, −*z*; (iii) −*x*+2, −*y*+1, −*z*+1.

## References

[bb1] Altomare, A., Cascarano, G., Giacovazzo, C., Guagliardi, A., Burla, M. C., Polidori, G. & Camalli, M. (1994). *J. Appl. Cryst.* **27**, 435.

[bb2] Binnemans, K. (2007). *Chem. Rev.* **107**, 2592–2614. 10.1021/cr050979c 17518503

[bb3] Brandner, A., Kitahara, T., Beare, N., Lin, C. K., Berry, M. T. & May, P. S. (2011). *Inorg. Chem.* **50**, 6509–6520. 10.1021/ic102538m 21675724

[bb4] Bruker (2007). *APEX2* and *SAINT* Bruker AXS Inc. Madison, Wisconsin, USA.

[bb5] Burrell, A. K., Del Sesto, R. E., Baker, S. N., McCleskey, T. M. & Baker, G. A. (2007). *Green Chem.* **9**, 449–454.

[bb6] Macrae, C. F., Bruno, I. J., Chisholm, J. A., Edgington, P. R., McCabe, P., Pidcock, E., Rodriguez-Monge, L., Taylor, R., van de Streek, J. & Wood, P. A. (2008). *J. Appl. Cryst.* **41**, 466–470.

[bb7] Matsumoto, K., Tsuda, T., Nohira, T., Hagiwara, R., Ito, Y. & Tamada, O. (2002). *Acta Cryst.* C**58**, m186–m187. 10.1107/s0108270101020601 11870297

[bb8] Pellens, M., Thijs, B., Van Hecke, K., Van Meervelt, L., Binnemans, K. & Nockemann, P. (2008). *Acta Cryst.* E**64**, m945. 10.1107/S1600536808018382 PMC2961749 21202797

[bb9] Samikkanu, S., Mellem, K., Berry, M. T. & May, P. S. (2007). *Inorg. Chem.* **46**, 7121–7128. 10.1021/ic070329m 17655287

[bb10] Sheldrick, G. M. (1996). *SADABS* Univeristy of Göttingen, Germany.

[bb11] Sheldrick, G. M. (2008). *Acta Cryst.* A**64**, 112–122. 10.1107/S0108767307043930 18156677

[bb12] Westrip, S. P. (2010). *J. Appl. Cryst.* **43**, 920–925.

